# Influence of diabetes on the efficacy of DL-3-n-butylphthalide in post-stroke cognitive impairment: a 12-month prospective cohort study

**DOI:** 10.3389/fnagi.2025.1649248

**Published:** 2025-11-05

**Authors:** Yuting Lu, Lifeng Wang, Juan Hao, Jingjing Song, Chenxi Fan, Xianjia Ning, Jinghua Wang, Yan Li

**Affiliations:** ^1^School of Basic Medical Sciences, Tianjin Medical University, Tianjin, China; ^2^Institute of Clinical Epidemiology and Evidence-Based Medicine, Tianjin Jizhou People’s Hospital, Tianjin, China; ^3^Department of Neurology, Tianjin Medical University General Hospital, Tianjin, China; ^4^Graduate School, Tianjin University of Traditional Chinese Medicine, Tianjin, China; ^5^Laboratory of Epidemiology, Tianjin Neurological Institute, Tianjin, China; ^6^Tianjin Neurological Institute, Key Laboratory of Post-Neuroinjury Neuro-repair and Regeneration in Central Nervous System, Ministry of Education and Tianjin City, Tianjin, China

**Keywords:** ischemic stroke, cognitive impairment, Dl-3-n-butylphthalide, diabetes mellitus, neuroprotection

## Abstract

**Objective:**

Diabetic chronic hyperglycaemia amplifies oxidative stress, microvascular injury, and insulin-resistant neuroinflammation, counteracting the pro-angiogenic, mitochondrial-protective, and anti-apoptotic effects of DL-3-n-butylphthalide (NBP). It remains unknown whether glycaemic status modulates the long-term cognitive benefits of NBP after ischaemic stroke (IS). This study compared 12-month efficacy of NBP on cognition between non-diabetic and diabetic patients with subacute IS.

**Methods:**

We conducted a community-based prospective cohort study involving 594 patients who had an ischemic stroke 1–6 months prior and no baseline cognitive impairment. Participants were assigned to either the NBP treatment group or the usual care group. MMSE scores were assessed at baseline and 12 months. The primary outcomes were ΔMMSE, its percentage change, and incident cognitive decline (≥3-point MMSE reduction). Separate multivariable regression analyses were conducted for non-diabetic and diabetic subgroups.

**Results:**

In non-diabetic patients (*n* = 360), NBP reduced the risk of cognitive decline by 45% (RR = 0.55, 95% CI 0.31–0.98, *p* = 0.043) and preserved language performance (*β* = −0.27, 95% CI –0.51 to −0.03). Among participants with diabetes (*n* = 234), NBP did not significantly lower decline incidence (RR = 0.63, 95% CI 0.33–1.19, *p* = 0.151), yet modestly improved orientation (*β* = −0.53, 95% CI –1.05 to −0.001, *p* = 0.045). Domain-specific analyses showed that NBP protected language in non-diabetic patients and orientation in diabetic patients (*p* < 0.05), while ΔMMSE was superior to control in both strata.

**Conclusion:**

In non-diabetic patients with subacute IS, NBP exerts more pronounced protective effects on overall cognition and language. In contrast, in diabetic patients, only a slight improvement in orientation is observed. Clinically, it is essential to prioritize optimization of diabetes management based on blood glucose control status before considering the addition of NBP. Further validation of these exploratory findings is warranted through larger-scale randomized trials.

## 1 Introduction

Stroke remains a leading cause of mortality and disability worldwide, with its incidence rising rapidly in low- and middle-income countries, posing a significant global health challenge (Prust et al., 2024). Ischemic stroke (IS) accounts for approximately 65.3% of all stroke events (GBD 2021 Nervous System Disorders Collaborators, 2024). Between 1990 and 2019, the absolute number of stroke cases increased by 70%, prevalence by 85%, and stroke-related deaths by 43% (GBD 2019 Stroke Collaborators, 2021). According to the Global Burden of Disease Study, stroke ranked as the leading cause of disability-adjusted life years (DALYs) globally from 2010 to 2021, and remains the foremost contributor in China (GBD 2021 Diseases and Injuries Collaborators, 2024).

Post-stroke cognitive impairment (PSCI) is one of the most common and disabling sequelae of stroke, affecting more than 70% of survivors (Rost et al., 2022). Approximately 38% of patients develop cognitive deficits within the first year after stroke (Sexton et al., 2019). Although some cases experience early recovery, up to one-third of stroke survivors progress to dementia within 5 years (El Husseini et al., 2023). In 2024, the projected cost of long-term care for individuals aged 65 years and older with cognitive impairment or dementia is estimated to exceed $360 billion in the United States alone (Alzheimer’s Association Report, 2024). These alarming statistics highlight the urgent need for effective secondary prevention strategies to preserve cognitive function in stroke survivors.

DL-3-n-butylphthalide (NBP), a compound derived from the seeds of *Apium graveolens*, has shown great potential in the treatment of IS (Tan et al., 2023). Its neuroprotective mechanisms include reducing oxidative stress, suppressing neuroinflammation, inhibiting apoptosis-related pathways, and promoting angiogenesis (Chen et al., 2020; Dai et al., 2023; Ge et al., 2024). Since its approval as an anti-ischemic agent in China in 2002, growing evidence has supported the role of NBP in improving cognitive function after stroke. A meta-analysis confirmed its beneficial effects on cognitive performance in patients with PSCI (Wang et al., 2024), and both short-term and long-term studies have demonstrated its efficacy in enhancing cognitive outcomes post-IS (Han et al., 2025; Wang et al., 2024; Yan et al., 2017).

Diabetes mellitus is a well-established risk factor for PSCI, independent of stroke severity and age (Filler et al., 2024). Elevated blood glucose levels in diabetes can lead to increased oxidative stress, which is known to disrupt the blood–brain barrier (BBB) and impair synaptic function in the hippocampus, a key region for memory and learning (Rom et al., 2019). This oxidative stress can also exacerbate neuroinflammation, further contributing to cognitive deficits. Additionally, diabetes-induced insulin resistance in the brain can disrupt insulin signaling pathways, which are crucial for maintaining neuronal health and synaptic plasticity (Ansari et al., 2023; Khalid et al., 2022). Notably, NBP may mitigate diabetes-related cognitive decline through mechanisms such as inhibiting caspase-3-mediated apoptosis and enhancing neurovascular protection (Tan et al., 2022). Preclinical studies have also demonstrated its neuroprotective effects in diabetic models (Tian et al., 2017). Accumulating evidence indicates that chronic hyperglycaemia undermines the pro-angiogenic and mitochondrial protective effects of NBP by intensifying oxidative stress, promoting advanced glycation end-product (AGE) deposition, and thickening the microvascular basement membrane (Khalid et al., 2022). Concomitantly, diabetes-induced injury within the fronto-striatal circuitry and parahippocampal region occurs early and is already extensive at the sub-acute stage, thereby narrowing the therapeutic window and attenuating the efficacy of NBP in diabetic patients (Moran et al., 2013). Furthermore, recent developments in nanotechnology-based delivery systems, such as solid lipid nanoparticles (SLNs), have shown promise in enhancing the brain targeting and bioavailability of neuroprotective agents like NBP (Bukke et al., 2024; Han et al., 2024). These novel formulations may help overcome pharmacokinetic challenges, especially in diabetic individuals who often exhibit altered drug metabolism and distribution.

Despite growing evidence supporting the use of NBP in IS and PSCI, a significant gap remains in the literature: no studies have conducted subgroup analyses based on diabetes status to elucidate the therapeutic effects of NBP in diabetic versus non-diabetic patients. Moreover, most existing research has been conducted in urban settings, with limited data available on rural populations, who often bear a higher disease burden and face greater barriers to post-stroke care. Diabetes mellitus was considered as a potential effect modifier in our study based on prior preclinical studies. Given these established pathophysiological links between diabetes and cognitive impairment, as well as the potential interactions with NBP mechanisms (e.g., inflammation, insulin resistance), we prioritized diabetes for subgroup analysis. This hypothesis-driven approach aims to elucidate whether glycemic status influences the efficacy of NBP in preventing PSCI.

Therefore, this study aimed to evaluate the differential effects of 12-month NBP therapy on cognitive function in diabetic and non-diabetic IS patients in a rural Chinese community. The findings will provide valuable evidence for optimizing NBP therapy and developing individualized strategies for the secondary prevention of cognitive impairment in stroke survivors.

## 2 Methods

### 2.1 Study design

This prospective community-based cohort study was conducted in Jizhou District, Tianjin. Participants were recruited and assigned to one of two groups using a 1:1 cluster sampling ratio, conducted over two phases. In the first phase (April to July 2021), eligible participants were enrolled in the NBP treatment group. In the second phase (October 2021 to March 2022), participants meeting the same inclusion criteria were recruited into the control group. The study was conducted in accordance with the Declaration of Helsinki and was approved by the Ethics Committee of the General Hospital of Tianjin Medical University (IRB2020-YX-056-02). All participants provided written informed consent. The study was registered with the Chinese Clinical Trial Registry (ChiCTR2000039118) on October 17, 2020.

### 2.2 Study participants

Eligible participants were adults aged 18 years or older, residing in Jizhou District, who had been diagnosed with non-cardiogenic IS by magnetic resonance imaging (MRI) within the past 6 months. All participants were functionally independent or had consistent caregiver support. Exclusion criteria included: hemorrhagic stroke, malignancy, coagulation disorders or cytopenia (platelets <100 × 10^9^/L), pregnancy, severe hepatic or renal dysfunction, heart failure, and participation in other clinical trials. All patients received standard post-stroke medical therapy, including antiplatelet, antihypertensive, antidiabetic, and/or lipid-lowering agents. The control group continued routine treatment, while the NBP group additionally received NBP soft capsules (0.2 g per dose, three times daily) for 12 consecutive months. To ensure medication adherence, we provided detailed instructions on medication use. For patients receiving NBP, we initially distributed a one-month supply of the medication upon enrollment, and subsequently replenished the supply every 3 months. During each replenishment, we collected empty bottles to monitor adherence, and all patients achieved a medication adherence rate of over 80%.

### 2.3 Data collection

Trained investigators conducted face-to-face interviews to collect demographic data (name, sex, age, and years of education), medical history (including diabetes, hypertension, hyperlipidemia, prior stroke, and coronary heart disease), and lifestyle factors (smoking and alcohol use). Physical examinations were performed using standardized protocols. Weight and height were measured to calculate body mass index (BMI), waist circumference (WC) was measured at the midpoint between the iliac crest and the lower rib, and hip circumference was measured at the widest part of the hips.

Blood pressure (BP) was measured using an automated sphygmomanometer after a 15-min rest period. BP was recorded in both arms, with two additional readings taken every 2 min, and the average value was used. All anthropometric and BP measurements were performed by the same examiner to minimize variability. After an overnight fast of at least 12 h, blood samples were collected to assess fasting blood glucose (FBG), glycosylated hemoglobin (HbA1c), total cholesterol (TC), triglycerides (TG), high-density lipoprotein (HDL), low-density lipoprotein (LDL), homocysteine (Hcy), and high-sensitivity C-reactive protein (hs-CRP).

### 2.4 Definitions and grouping

BMI was calculated as weight (kg) divided by height squared (m^2^) and categorized as underweight (<18.5 kg/m^2^), normal weight (18.5–23.9 kg/m^2^), or overweight (24–27.9 kg/m^2^) based on Chinese standards (Chen et al., 2023). Diabetes was defined as any of the following: HbA1c ≥ 6.5%, FPG ≥ 126 mg/dL (7.0 mmol/L), 2-h post-OGTT glucose ≥200 mg/dL (11.1 mmol/L), use of hypoglycemic medication, or self-reported history of diabetes (American Diabetes Association Professional Practice Committee, 2024). Participants were grouped into diabetic and non-diabetic subgroups based on baseline status.

### 2.5 Assessment of cognitive function and outcomes

Cognitive function was evaluated at baseline and after 12 months using the Mini-Mental State Examination (MMSE), which assesses orientation, memory, attention and calculation, recall, and language (Chun et al., 2021). Scores range from 0 to 30, with lower scores indicating poorer cognitive function. Based on a population-based study in elderly Chinese individuals, education-specific MMSE cutoffs were applied to define cognitive impairment: ≤17 for illiterate individuals, ≤20 for those with ≤6 years of education, and ≤24 for those with >6 years of education (Li et al., 2016).

Primary outcomes included the change in MMSE score (ΔMMSE = baseline score minus 12-month score), the percentage change, and the incidence of cognitive decline. Cognitive decline was defined as a ≥ 3-point decrease in MMSE score (Han et al., 2025). Because ΔMMSE intrinsically adjusts for baseline differences, baseline MMSE was not entered as an additional covariate in the between-group comparisons. Secondary outcomes included changes and percentage changes in individual cognitive domains (orientation, memory, language, etc.) in both diabetic and non-diabetic groups.

### 2.6 Statistical analysis

*A priori* power analysis was conducted using G*Power 3.1 to determine the minimum sample size required to detect a moderate effect size (Cohen’s *d* = 0.5) with a significance level (*α*) of 0.05 and a statistical power of 0.80 (1 − *β*). The calculation indicated that at least 128 participants per group (256 total) would be required for sufficient power in detecting differences in cognitive outcomes. Given our actual sample size (594 participants, 301 in the NBP group and 293 in the control group), the study was adequately powered to detect clinically meaningful effects. Continuous variables were reported as mean ± standard deviation (SD) or median with interquartile range (IQR) and compared using Student’s t-test, ANOVA, or Mann–Whitney U test, as appropriate. Categorical variables were presented as frequencies and percentages and compared using chi-square tests. Standardized mean differences (SMDs) were computed to quantify baseline imbalances between groups; an absolute SMD > 0.20 indicated a clinically meaningful imbalance and, together with established confounders, was adjusted for in all multivariable models. The selection of variables for univariate analysis was based on their clinical significance, including age, BMI, medical history, lipid levels, and inflammatory markers. These variables were chosen due to their established associations with cognitive outcomes in IS patients. Variables with *p* < 0.05 in the univariate analysis were included in the linear regression models, while those with *p* < 0.2 were included in the logistic regression models, to ensure comprehensive adjustment for potential confounders. Multivariate logistic regression was used to assess the association between NBP treatment and the risk of cognitive decline, while multivariate linear regression was used to analyze changes in MMSE scores and cognitive domain performance. Results are expressed as approximate adjusted risk ratios (RR), regression coefficients (*β*), and 95% confidence intervals (CIs). A two-tailed *p* value <0.05 was considered statistically significant. All analyses were performed using SPSS (v2.7, IBM, USA) and GraphPad Prism (v10.2.3, San Diego, CA, USA).

## 3 Results

### 3.1 Study population

**Figure 1:**
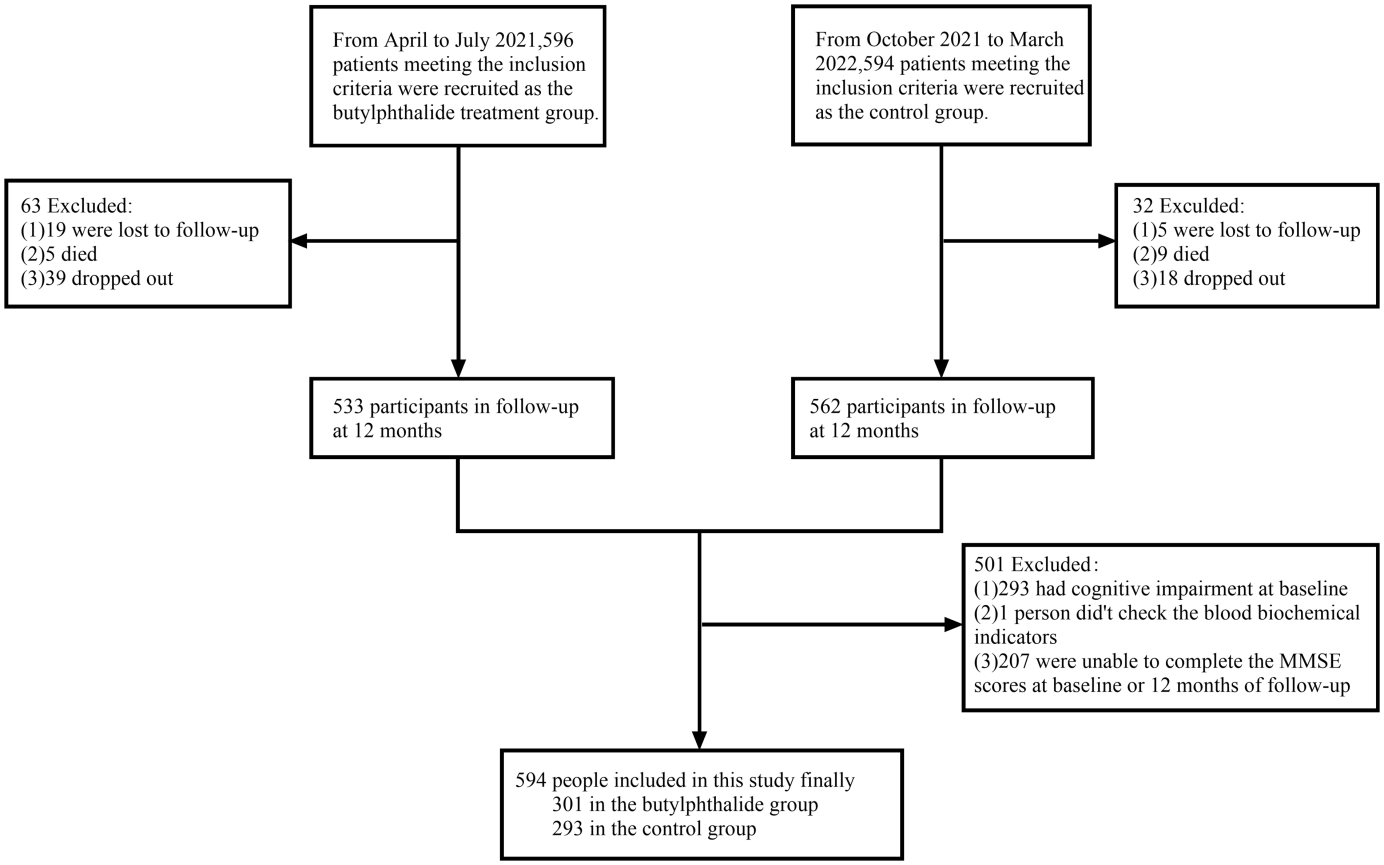
Flow chat of participants selection. This figure showed that from April 2021 to March 2022, 1,190 ischemic stroke patients were screened. A total of 596 and 594 participants were initially enrolled in the NBP treatment group and control group, respectively. After exclusions (lost to follow-up, death, dropout, and baseline cognitive impairment), 594 participants (301 in the NBP group and 293 in the control group) were included in the final analysis.

Between April 2021 and March 2022, a total of 1,190 IS patients were screened for eligibility. Among them, 596 participants were allocated to the NBP treatment group and 594 to the control group. After 1 year of follow-up, excluding individuals with incomplete cognitive data and those with cognitive impairment at baseline, 594 participants were included in the final analysis: 301 in the NBP group and 293 in the control group (Figure 1).

### 3.2 Baseline characteristics

The study population consisted of 418 males (70.4%) and 176 females (29.6%), with a mean age of 61.73 ± 8.54 years and a mean education level of 7.23 ± 3.45 years. The overall prevalence of diabetes was 39.4%. The median MMSE score at baseline was 27 (interquartile range: 25–29). Baseline demographic and clinical characteristics are summarized in Table 1.

**Table 1:** Demographic characteristics among all participants.

Characteristics	Control	Butylphthalide	Total	SMD
Total, *n* (%)	293 (49.3)	301 (50.7)	594 (100.0)	
Gender, *n* (%)				
Men	210 (71.7)	208 (69.1)	418 (70.4)	
Women	83 (28.3)	93 (30.9)	176 (29.6)	
Age, mean (SD), years	62.17 (8.21)	61.30 (8.84)	61.73 (8.54)	8.54
Age group, *n* (%)				
<60 years old	110 (37.5)	126 (41.9)	236 (39.7)	
≥60 years old	183 (62.5)	175 (58.1)	358 (60.3)	
Education years, mean (SD), years^*^	7.07 (3.41)	7.39 (3.48)	7.23 (3.45)	3.45
Education group, *n* (%)^*^				
Illiterate	18 (6.2)	17 (5.6)	35 (5.9)	
Primary school	116 (39.7)	106 (35.2)	222 (37.4)	
Junior school	114 (39.0)	115 (38.2)	229 (38.6)	
High school and above	44 (15.1)	63 (20.9)	107 (18.0)	
WHR, mean (SD)^*^	0.93 (0.08)	0.95 (0.08)	0.94 (0.08)	0.08
BMI, mean (SD), kg/m^2^	26.63 (3.14)	26.89 (3.73)	26.76 (3.45)	3.45
BMI groups, *n* (%)				
Normal or underweight	58 (19.8)	65 (21.6)	123 (20.7)	
Overweight	145 (49.5)	129 (42.9)	274 (46.1)	
Obesity	90 (30.7)	107 (35.5)	197 (33.2)	
Smoking history, *n* (%)				
Never smoking	80 (27.3)	57 (18.9)	137 (23.1)	
Current smoking	102 (34.8)	130 (43.2)	232 (39.1)	
Ever smoking	111 (37.9)	114 (37.9)	225 (37.9)	
Drinking history, *n* (%)				
Never drinking	67 (22.9)	33 (11.0)	100 (16.8)	
Current drinking	125 (42.7)	141 (46.8)	266 (44.8)	
Ever drinking	101 (34.5)	127 (42.2)	228 (38.4)	
Hypertension history, *n* (%)				
No	86 (29.4)	66 (21.9)	152 (25.6)	
Yes	207 (70.6)	235 (78.1)	442 (74.4)	
Diabetes, *n* (%)				
No	181 (61.8)	179 (59.5)	360 (60.6)	
Yes	112 (38.2)	122 (40.5)	234 (39.4)	
Hyperlipidemia history, *n* (%)				
No	178 (60.8)	160 (53.2)	338 (56.9)	
Yes	115 (39.2)	141 (46.8)	256 (43.1)	
CHD, *n* (%)				
No	263 (89.8)	261 (86.7)	524 (88.2)	
Yes	30 (10.2)	40 (13.3)	70 (11.8)	
SBP, mean (SD), mmHg^*^	154.92 (20.88)	151.32 (20.31)	153.10 (20.65)	20.59
DBP, mean (SD), mmHg^*^	94.24 (11.61)	94.99 (11.34)	94.62 (11.47)	11.47
FBG, mean (SD), mmol/L	6.74 (2.67)	6.90 (2.21)	6.83 (2.24)	2.45
TC, mean (SD), mmol/L	4.44 (1.12)	4.34 (1.05)	4.39 (1.08)	1.09
TG, mean (SD), mmol/L	1.58 (1.30)	1.59 (1.04)	1.58 (1.18)	1.18
HDL, mean (SD), mmol/L	1.24 (0.29)	1.20 (0.28)	1.22 (0.29)	0.28
LDL, mean (SD), mmol/L	2.55 (0.90)	2.49 (0.86)	2.52 (0.88)	0.88
Hcy, mean (SD), mmol/L	16.62 (10.99)	17.51 (12.68)	17.07 (11.87)	11.87
hs-CRP, median (IQR), mg/L	1.40 (0.75–2.89)	1.39 (0.59–2.77)	1.40 (0.68–2.85)	5.22
mRS score, median (IQR)	1 (0–1)	1 (0–1)	1 (0–1)	0.90
MMSE score, median (IQR)	27 (25–29)	27 (25–28)	27 (25–29)	2.77
Time of inclusion in the study, month	3 (3–10)	4 (4–5)	4 (3–7)	2.62

(1) ^*^ indicates the missing value, including 1 case of education years, 3 cases of WHR, 1 case of SBP and DBP deletion. The proportion of missing values is less than 0.1%.

(2) Continuous variables are expressed as mean (SD) or median (IQR). SMD, Standardized Mean Difference; WHR, Waist-to-Hip Ratio; BMI, body mass index; CHD, coronary heart disease; SBP, systolic blood pressure; DBP, diastolic blood pressure; FPG, fasting plasma glucose; TG, triglycerides; TC, total cholesterol; HDL, High density lipoprotein; LDL, Low density lipoprotein; Hcy, homcysteine; hs-CRP, high-sensitivity C-reactive protein; MMSE, Mini-Mental State Examination; mRS, modified Rankin Scale.

### 3.3 Effect of NBP on MMSE score change and percentage

Although the study was *a priori* stratified by metabolic status, we further examined the NBP × diabetes interaction term in the overall model. The interaction was not statistically significant (*p* = 0.838), indicating no appreciable modification of the NBP effect by diabetes at the population level. Results from the subgroup analyses showed that, in univariate comparisons, both the absolute MMSE score change (ΔMMSE) and its percentage were significantly lower in the NBP group compared to the control group (*p* < 0.05), suggesting a protective effect of NBP on cognitive decline (Table 2). After adjusting for confounders, NBP treatment was associated with a significantly smaller ΔMMSE and percentage change in both diabetic and non-diabetic subgroups.

**Table 2:** Univariate analysis of risk factors for MMSE D-value and percentage in diabetics and non-diabetics.

Characteristics	Diabetics				Non-diabetics			
	MMSE D-value	*p* value	MMSE D-value percentage	*p* value	MMSE D-value	*p* value	MMSE D-value percentage	*p* value
Treatment, *n* (%)		**0.036**		**0.033**		**0.005**		**0.008**
Butylphthalide	0.47 (3.22)		0.01 (0.13)		0.08 (2.64)		0.002 (0.11)	
Control	1.55 (4.49)		0.06 (0.20)		1.05 (3.73)		0.04 (0.16)	
Gender, *n* (%)		0.374		0.363		0.679		0.511
Men	0.83 (3.58)		0.03 (0.15)		0.52 (2.89)		0.02 (0.11)	
Women	1.39 (4.64)		0.06 (0.21)		0.69 (3.99)		0.03 (0.18)	
Age, years	0.05 (−0.01, 0.11)	0.091	0.002 (0.000, 0.005)	0.073	0.08 (0.04, 0.12)	**<0.001**	0.003 (0.002, 0.01)	**<0.001**
Age group, *n* (%)		**0.025**		**0.014**		**0.003**		**0.002**
<60 years	0.33 (3.14)		0.01 (0.12)		−0.01 (2.40)		−0.004 (0.09)	
≥60 years	1.43 (4.30)		0.06 (0.19)		0.95 (3.67)		0.04 (0.16)	
Education years, years	−0.16 (−0.31, -0.02)	**0.029**	−0.01 (−0.01, -0.002)	**0.013**	−0.16 (−0.26, -0.07)	**0.001**	−0.01 (−0.01, -0.003)	**<0.001**
Educational level, *n* (%)		0.188		0.061		**0.044**		**0.042**
Illiterate	2.93 (6.84)		0.14 (0.35)		2.90 (5.87)		0.13 (0.28)	
Primary school	1.14 (4.48)		0.04 (0.19)		0.75 (3.86)		0.03 (0.16)	
Junior school	0.71 (3.18)		0.02 (0.12)		0.38 (2.38)		0.01 (0.09)	
High school and above	0.55 (2.10)		0.02 (0.07)		−0.03 (1.95)		−0.003 (0.07)	
WHR	1.27 (−4.79, 7.32)	0.681	0.03 (−0.23, 0.30)	0.819	−0.03 (−0.68, 0.62)	0.934	−0.002 (−0.03, 0.03)	0.888
BMI, kg/m^2^	−0.09 (−0.24, 0.06)	0.239	−0.004 (−0.01, 0.002)	0.209	−0.04 (−0.14, 0.06)	0.425	−0.002 (−0.01, 0.002)	0.391
BMI groups, *n* (%)		0.141		0.193		0.529		0.432
Normal, underweight	0.34 (3.90)		0.02 (0.17)		0.75 (3.25)		0.03 (0.13)	
Overweight	1.52 (4.09)		0.06 (0.17)		0.66 (3.42)		0.03 (0.15)	
Obesity	0.58 (3.64)		0.02 (0.15)		0.27 (3.01)		0.01 (0.12)	
Smoking status, *n* (%)		0.270		0.314		0.629		0.613
Never smoking	1.48 (4.51)		0.06 (0.19)		0.75 (2.76)		0.03 (0.11)	
Current smoking	1.25 (4.30)		0.05 (0.19)		0.65 (3.73)		0.03 (0.17)	
Ever smoking	0.51 (3.12)		0.02 (0.13)		0.35 (3.05)		0.01 (0.12)	
Drinking status, *n* (%)		0.298		0.381		0.761		0.849
Never drinking	0.61 (3.13)		0.02 (0.12)		0.84 (2.53)		0.03 (0.10)	
Current drinking	1.42 (4.17)		0.05 (0.18)		0.51 (3.58)		0.02 (0.16)	
Ever drinking	0.53 (3.83)		0.02 (0.16)		0.51 (3.21)		0.02 (0.13)	
Hypertension, *n* (%)		0.955		0.993		0.564		0.581
No	0.96 (3.98)		0.04 (0.17)		0.74 (3.64)		0.03 (0.16)	
Yes	0.99 (3.90)		0.04 (0.17)		0.50 (3.10)		0.02 (0.13)	
Hyperlipidemia, *n* (%)		0.653		0.542		0.083		0.061
No	1.10 (4.01)		0.04 (0.18)		0.78 (3.62)		0.03 (0.16)	
Yes	0.87 (3.82)		0.03 (0.16)		0.22 (2.55)		0.01 (0.10)	
CHD, *n* (%)		0.282		0.369		0.707		0.649
No	0.88 (3.84)		0.03 (0.16)		0.54 (3.15)		0.02 (0.14)	
Yes	1.67 (4.32)		0.07 (0.20)		0.81 (4.18)		0.03 (0.17)	
SBP, mmHg	0.004 (−0.02, 0.03)	0.781	0.0002 (−0.001, 0.001)	0.725	0.03 (0.01, 0.04)	**0.001**	0.001 (0.0005, 0.002)	**<0.001**
DBP, mmHg	−0.02 (−0.06, 0.03)	0.419	−0.001 (−0.003, 0.001)	0.463	0.04 (0.004, 0.07)	**0.025**	0.002 (0.0002, 0.003)	**0.021**
FBG, mmol/L	−0.01 (−0.20, 0.19)	0.949	−0.001 (−0.01, 0.01)	0.785	0.10 (−0.54, 0.73)	0.294	0.01 (−0.02, 0.03)	0.588
TC, mmol/L	−0.03 (−0.49, 0.44)	0.912	0.0004 (−0.02, 0.02)	0.969	0.33 (0.01, 0.65)	**0.043**	0.01 (0.0003, 0.03)	**0.044**
TG, mmol/L	−0.26 (−0.71, 0.20)	0.265	−0.01 (−0.03, 0.01)	0.243	−0.04 (−0.32, 0.24)	0.798	−0.001 (−0.01, 0.01)	0.929
HDL, mmol/L	3.47 (1.61, 5.33)	**<0.001**	0.15 (0.07, 0.23)	**<0.001**	0.53 (−0.65, 1.70)	0.379	0.02 (−0.03, 0.07)	0.463
LDL, mmol/L	−0.31 (−0.92, 0.30)	0.313	−0.01 (−0.04, 0.02)	0.433	0.40 (0.03, 0.78)	**0.035**	0.02 (0.001, 0.03)	**0.042**
Hcy, mmol/L	−0.01 (−0.06, 0.03)	0.596	0.0004 (−0.002, 0.001)	0.663	0.01 (−0.02, 0.04)	0.552	0.0003 (−0.001, 0.002)	0.567
Hs-CRP, mg/L	−0.03 (−0.11, 0.04)	0.379	−0.001 (−0.005, 0.002)	0.372	−0.05 (−0.13, 0.04)	0.305	−0.002 (−0.01, 0.002)	0.339
Time of inclusion in the study, month	−0.03 (−0.22, 0.16)	0.773	−0.001 (−0.01, 0.01)	0.760	−0.03 (−0.15, 0.10)	0.659	−0.001 (−0.007, 0.004)	0.591

(1) MMSE scores and cognitive domain scores are expressed as mean (SD) or *β* (95%CI).

(2) Bold fonts indicate *p* < 0.05, with significant differences in statistical results.

(3) WHR, Waist-to-Hip Ratio; BMI, body mass index; CHD, coronary heart disease; SBP, systolic blood pressure; DBP, diastolic blood pressure; FPG, fasting plasma glucose; TG, triglycerides; TC, total cholesterol; HDL, High density lipoprotein; LDL, Low density lipoprotein; Hcy, homcysteine; hs-CRP, high-sensitivity C-reactive protein.

**Figure 2:**
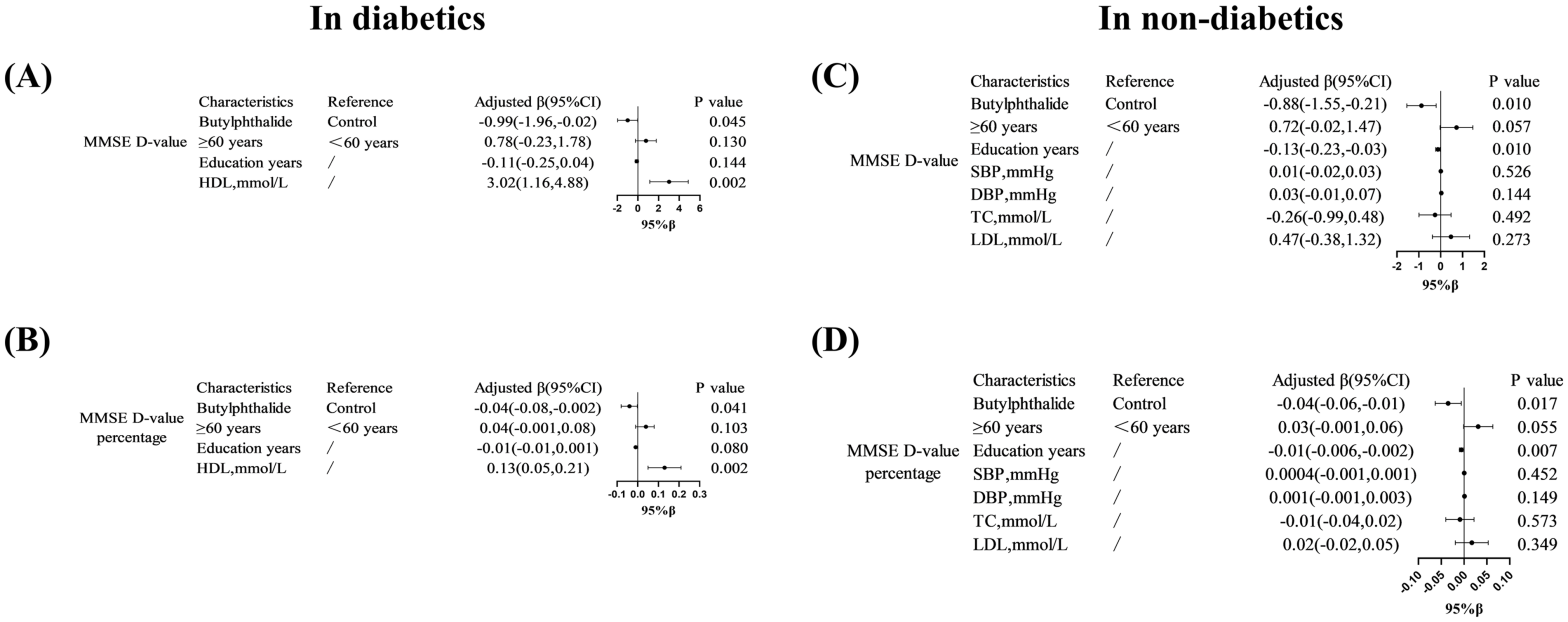
Multivariate linear regression analysis of MMSE score changes in diabetic patients treated with NBP. **(A–D)** Showed that associations between NBP treatment and MMSE score changes (ΔMMSE) in diabetic subgroups. Variables included education years and HDL levels. Results are presented as adjusted β coefficients (95% CI) and *p*-values. Significance levels: *p* < 0.05.

**Figure 3:**
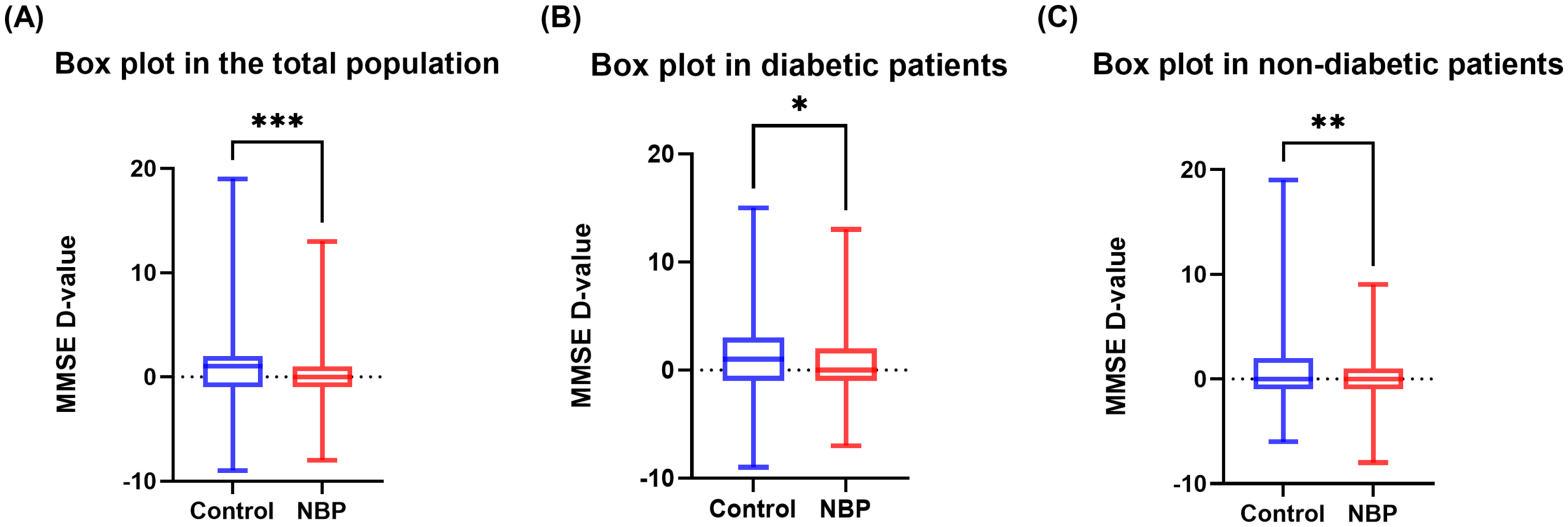
Box plots of MMSE change. **(A)** 12-month MMSE change in the total population; **(B)** diabetic subgroup; **(C)** non-diabetic subgroup. Box boundaries mark the 25th and 75th percentiles, the line indicates the median, whiskers extend to 1.5 × interquartile range, and dots denote outliers. Asterisks indicate between-group *p* values: *p < 0.05, **p < 0.01, ***p < 0.001.

In the diabetic subgroup, the adjusted regression coefficients for ΔMMSE and its percentage were *β* = −0.99 (95% CI, −1.96 to −0.02, *p* = 0.045) and *β* = −0.04 (95% CI, −0.08 to −0.002, *p* = 0.041), respectively. Similarly, in non-diabetic patients, NBP treatment was significantly associated with a smaller cognitive decline: *β* = −0.88 (95% CI, −1.55 to −0.21, *p* = 0.010) and *β* = −0.04 (95% CI, −0.06 to −0.01, *p* = 0.017) (Figure 2). Figure 3 illustrated the distribution of 12-month MMSE change (Figure 3). In the total population, the median ΔMMSE was significantly higher in the NBP group than in controls (*p* < 0.01, Figure 3A). The diabetic subgroup showed the same trend (*p* < 0.05, Figure 3B), while the non-diabetic subgroup exhibited the largest improvement (*p* < 0.001, Figure 3C), consistent with the primary analyses.

### 3.4 Effect of NBP on risk of cognitive decline

The NBP × diabetes interaction was not statistically significant for the overall incidence of cognitive decline (RR = 0.935, 95% CI: 0.57–1.54, *p* = 0.790). After stratification by glycaemic status, univariate analyses revealed that the incidence of cognitive decline was significantly lower in the NBP group than in the control group (14.5% vs. 23.2%, *p* = 0.035). However, this difference was not statistically significant in the diabetic subgroup (19.7% vs. 26.8%, *p* = 0.197). Variables such as age, education, alcohol consumption, systolic blood pressure (SBP), and HDL-C were found to be associated with cognitive decline in univariate analysis (Table 3).

**Table 3:** Univariate analysis of risk factors for cognitive decline in diabetics and non-diabetics.

Characteristics	Diabetics			Non-diabetics		
	No cognitive decline	Cognitive decline	*p* value	No cognitive decline	Cognitive decline	*p* value
Treatment, *n* (%)			0.197			**0.035**
Butylphthalide	98 (80.3)	24 (19.7)		153 (85.5)	26 (14.5)	
Control	82 (73.2)	30 (26.8)		139 (76.8)	42 (23.2)	
Gender, *n* (%)			0.100			0.069
Men	134 (79.8)	34 (20.2)		209 (83.6)	41 (16.4)	
Women	46 (69.7)	20 (30.3)		83 (75.5)	27 (24.5)	
Age, years	61.27 (8.90)	63.22 (7.77)	0.148	60.96 (8.50)	65.04 (7.55)	**<0.001**
Age group, *n* (%)			0.138			**0.031**
<60 years	77 (81.9)	17 (18.1)		123 (86.6)	19 (13.4)	
≥60 years	103 (73.6)	37 (26.4)		169 (77.5)	49 (22.5)	
Education years, years	7.34 (3.38)	6.63 (3.67)		7.62 (3.38)	5.75 (3.35)	**<0.001**
Educational level, *n* (%)			0.547			**0.004**
Illiterate	10 (66.7)	5 (33.3)		12 (60.0)	8 (40.0)	
Primary school	65 (73.9)	23 (26.1)		102 (76.1)	32 (23.9)	
Junior school	73 (80.2)	18 (19.8)		116 (84.1)	22 (15.9)	
High school and above	32 (80.0)	8 (20.0)		61 (91.0)	6 (9.0)	
WHR	0.95 (0.08)	0.95 (0.10)	0.908	0.97 (0.58)	0.91 (0.07)	0.446
BMI, kg/m^2^	27.56 (3.55)	26.78 (2.79)	0.095	26.41 (3.40)	26.14 (3.55)	0.558
BMI groups, *n* (%)			0.099			0.816
Normal, underweight	27 (84.4)	5 (15.6)		72 (79.1)	19 (20.9)	
Overweight	77 (70.6)	32 (29.4)		134 (81.2)	31 (18.8)	
Obesity	76 (81.7)	17 (18.3)		86 (82.7)	18 (17.3)	
Smoking status, *n* (%)			0.197			0.051
Never smoking	35 (76.1)	11 (23.9)		73 (80.2)	18 (19.8)	
Current smoking	65 (71.4)	26 (28.6)		107 (75.9)	34 (24.1)	
Ever smoking	80 (82.5)	17 (17.5)		112 (87.5)	16 (12.5)	
Drinking status, *n* (%)			**0.034**			0.404
Never drinking	27 (81.8)	6 (18.2)		56 (83.6)	11 (16.4)	
Current drinking	74 (69.2)	33 (30.8)		124 (78.0)	35 (22.0)	
Ever drinking	79 (84.0)	15 (16.0)		112 (83.6)	22 (16.4)	
Hypertension, *n* (%)			0.618			0.892
No	39 (79.6)	10 (20.4)		84 (81.6)	19 (18.4)	
Yes	141 (76.2)	44 (23.8)		208 (80.9)	49 (19.1)	
Hyperlipidemia, *n* (%)			0.445			0.056
No	86 (74.8)	29 (25.2)		174 (78.0)	49 (22.0)	
Yes	94 (79.0)	25 (21.0)		118 (86.1)	19 (13.9)	
CHD, *n* (%)			0.537			0.654
No	156 (77.6)	45 (22.4)		263 (81.4)	60 (18.6)	
Yes	24 (72.7)	9 (27.3)		29 (78.4)	8 (21.6)	
SBP, mmHg	152.84 (20.41)	153.31 (19.33)	0.879	152.13 (21.14)	157.82 (19.97)	**0.046**
DBP, mmHg	93.89 (12.18)	92.42 (10.54)	0.423	95.07 (10.94)	96.40 (12.26)	0.382
FBG, mmol/L	8.65 (2.57)	8.73 (2.64)	0.847	5.64 (0.54)	5.58 (0.51)	0.389
TC, mmol/L	4.19 (1.10)	4.20 (1.07)	0.951	4.48 (1.06)	4.68 (1.04)	0.148
TG, mmol/L	1.67 (1.06)	1.53 (1.32)	0.403	1.56 (1.29)	1.52 (1.29)	0.810
HDL, mmol/L	1.11 (0.23)	1.25 (0.33)	**0.006**	1.27 (0.29)	1.29 (0.26)	0.577
LDL, mmol/L	2.41 (0.81)	2.32 (0.88)	0.492	2.57 (0.90)	2.77 (0.90)	0.094
Hcy, mmol/L	16.26 (11.28)	16.54 (12.17)	0.876	17.67 (12.10)	17.07 (12.27)	0.715
Hs-CRP, mg/L	1.50 (0.76–3.71)	1.27 (0.82–2.80)	0.446	1.29 (0.65–2.82)	1.44 (0.67–2.37)	0.521
Time of inclusion in the study, month	4.5 (3.5–7)	4 (3–6)	0.449	4 (4–7)	4 (3–7)	0.441

(1) Bold fonts indicate *p* < 0.05, with significant differences in statistical result.

(2) Continuous variables are expressed as mean (SD) or median (IQR).

(3) WHR, Waist-to-Hip Ratio; BMI, body mass index; CHD, coronary heart disease; SBP, systolic blood pressure; DBP, diastolic blood pressure; FPG, fasting plasma glucose; TG, triglycerides; TC, total cholesterol; HDL, High density lipoprotein; LDL, Low density lipoprotein; Hcy, homcysteine; hs-CRP, high-sensitivity C-reactive protein.

**Figure 4:**
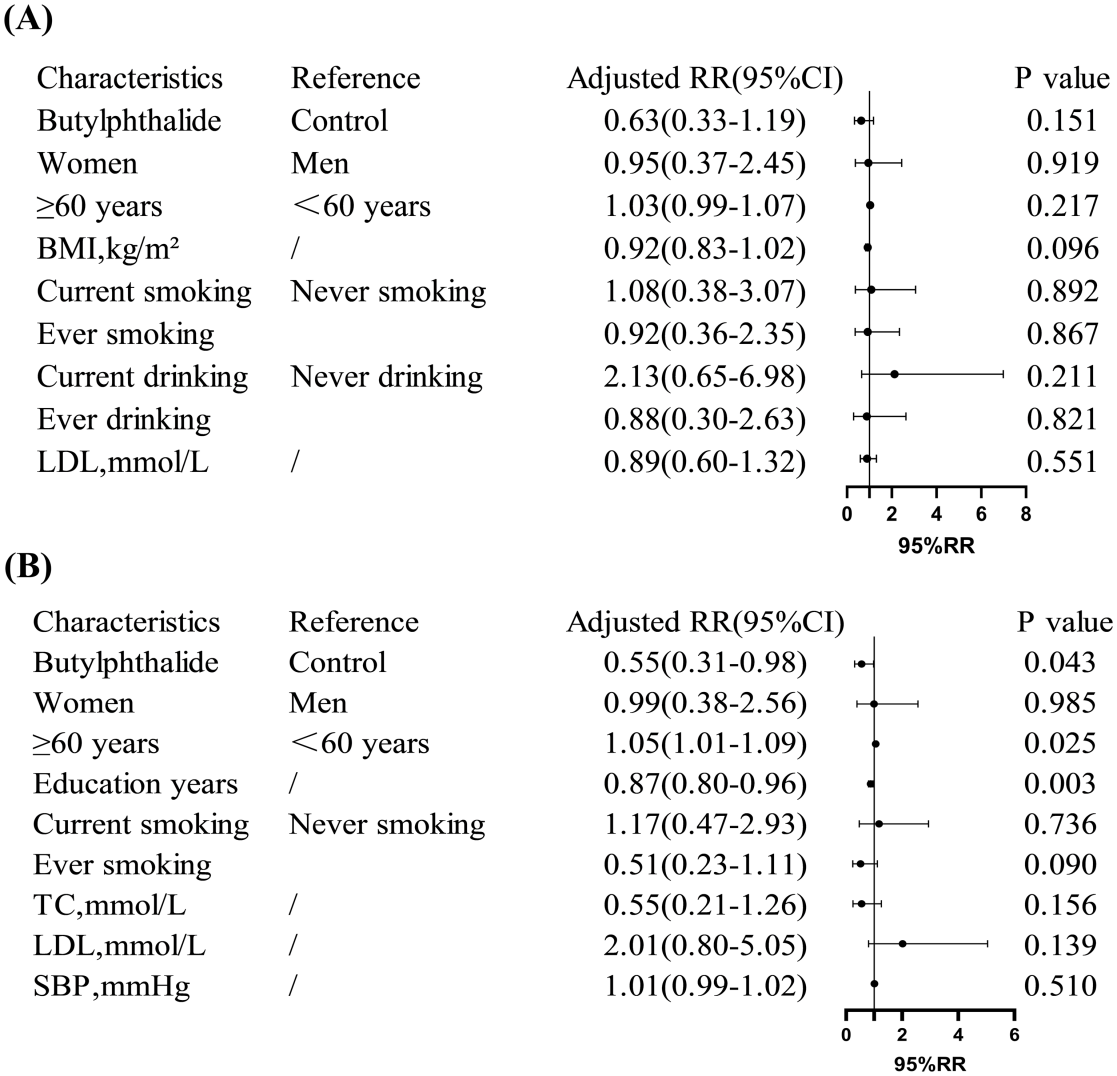
Risk ratios (RR) for cognitive decline in diabetic and non-diabetic subgroups. **(A)** Showed that in diabetic patients, NBP treatment showed no significant reduction in cognitive decline risk (adjusted RR = 0.63, 95% CI: 0.33, 1.19). **(B)** Showed that in non-diabetic patients, NBP significantly reduced the risk (adjusted RR = 0.55, 95% CI: 0.31, 0.98). Covariates included age, education years, and smoking status.

Multivariate logistic regression indicated that NBP treatment significantly reduced the risk of cognitive decline in non-diabetic patients (RR = 0.55, 95% CI: 0.31–0.98, *p* = 0.043), but not in diabetic patients (RR = 0.63, 95% CI: 0.33–1.19, *p* = 0.151). In addition, among non-diabetic patients, age ≥60 years was associated with a 5% increased risk of cognitive decline (*p* = 0.025), while each additional year of education was associated with a 13% reduction in risk (*p* = 0.003) (Figure 4).

### 3.5 Effect of NBP on specific cognitive domains

Analysis of changes in specific cognitive domains revealed differential effects of NBP treatment between diabetic and non-diabetic participants. In the diabetic group, NBP significantly attenuated the decline in orientation scores compared with the control group (mean Δ = 0.05 ± 1.76 vs. 0.60 ± 2.32, *p* = 0.042), while no significant effect was observed on language scores (*p* > 0.05) (Supplementary Table 1).

In contrast, in the non-diabetic group, NBP treatment significantly preserved language function (mean Δ = 0.00 ± 1.09 vs. 0.30 ± 1.22, *p* = 0.013; percentage change: −0.01 ± 0.16 vs. 0.03 ± 0.17, *p* = 0.015), while orientation scores did not differ significantly (Supplementary Table 2). No significant differences were observed in other domains (attention/calculation, memory, or recall) in either group (Supplementary Table 3).

**Figure 5:**
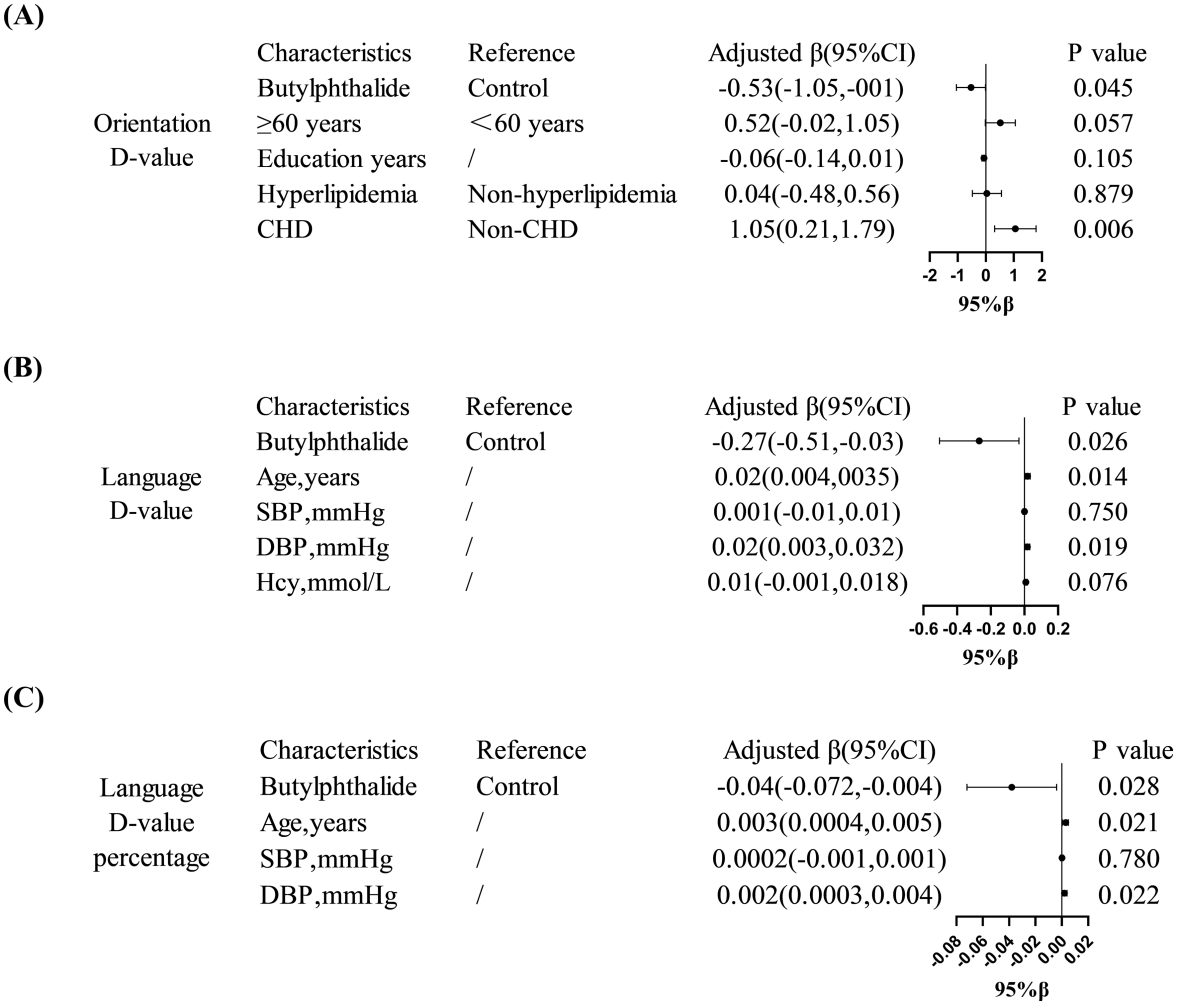
Domain-specific cognitive effects of NBP treatment. **(A)** Showed that in diabetic patients, NBP improved orientation ability (adjusted *β* = −0.53, 95% CI: −1.05, −0.001). **(B,C)** Showed that in non-diabetic patients, NBP preserved language function (adjusted *β* = −0.27, 95% CI: −0.51, −0.03).

Multivariate linear regression confirmed these findings: NBP was associated with better orientation ability in diabetic patients (*β* = −0.53, 95% CI: −1.05 to −0.001, *p* = 0.045) and better language function in non-diabetic patients (*β* = −0.27, 95% CI: −0.51 to −0.03, *p* = 0.026). Furthermore, in the diabetic subgroup, a history of coronary heart disease was positively correlated with a greater decline in orientation (*β* = 1.05, 95% CI: 0.21–1.79, *p* = 0.006). In non-diabetic patients, both age and diastolic blood pressure (DBP) were significantly associated with language decline (*p* < 0.05) (Figure 5).

## 4 Discussion

The primary objective of this study was to investigate the differential effects of NBP on cognitive impairment in IS patients with and without diabetes, with the goal of better evaluating its therapeutic potential in diverse patient populations. To our knowledge, this is the first study to report the long-term (12-month) effects of NBP treatment on cognitive outcomes in IS patients stratified by glycemic status. Our findings suggest that NBP is more effective in preventing cognitive decline in non-diabetic patients compared to those with diabetes. Furthermore, the cognitive domains influenced by NBP appear to differ between the two groups: NBP primarily preserved orientation in diabetic patients, whereas it was beneficial to language function in non-diabetic patients. These results highlight NBP’s promise as a targeted neuroprotective therapy and suggest that its mechanisms of action may vary depending on metabolic context.

NBP confers neuroprotection by upregulating vascular endothelial growth factor (VEGF)/CD31 to enhance angiogenesis and by dampening oxidative/nitrosative stress and pro-inflammatory pathways, thereby reducing infarct volume, improving neurological function, cerebral perfusion and mitochondrial fusion, and optimizing stroke outcome (Chen et al., 2020; Dai et al., 2023; Zhu et al., 2024). Meta-analyses confirm its efficacy, alone or combined, in ameliorating post-stroke cognition, with clinical trials showing benefits within 1 month that persist for 12 months (Fan et al., 2021; Han et al., 2025; Wang et al., 2024; Yan et al., 2017).

Our findings are consistent with this prior evidence, showing a beneficial effect of NBP on ΔMMSE regardless of diabetic status. However, its ability to prevent binary-defined cognitive decline was more pronounced in non-diabetic patients. This discrepancy may reflect the greater vulnerability of diabetic patients to chronic metabolic injury, which may blunt the cognitive benefits of NBP over the 12-month treatment period. Diabetes-induced hyperglycemia impairs hippocampal function by disrupting insulin signaling pathways, leading to synaptic dysfunction and memory deficits (Ansari et al., 2023). Additionally, the accumulation of AGEs triggers oxidative stress and inflammation, further damaging neuronal structures and exacerbating cognitive decline (Khalid et al., 2022). Neuroinflammation also compromises the integrity of the BBB, creating a vicious cycle that amplifies memory loss (Rom et al., 2019). Microvascular damage, another common complication of diabetes, can reduce cerebral blood flow and oxygen delivery to the brain, further impairing cognitive function (De Silva and Faraci, 2016). These mechanisms may explain why NBP’s efficacy in preventing cognitive decline was attenuated in diabetic patients compared to non-diabetic patients in our study. While our study administered NBP for 12 months, it is possible that a longer treatment duration is needed to observe more significant cognitive benefits in diabetic patients. Notably, although we observed an improvement in MMSE score changes, the magnitude of the difference fell short of the established minimal clinically important difference, indicating only a therapeutic trend rather than confirmed clinical benefit. Importantly, on the binary outcome of cognitive decline, we detected a protective effect of NBP exclusively in non-diabetic participants, suggesting that individuals with fewer baseline metabolic disturbances derived greater benefit. These findings underscore the need for longer-term follow-up to validate the robustness of the observed protection and to determine whether the early functional gains translate into a sustained reduction in dementia risk. While our study provides valuable insights into the differential effects of NBP on cognitive outcomes in diabetic and non-diabetic ischemic stroke patients, it is important to note that detailed glycemic control data were not specifically collected during the 12-month follow-up period. However, our study design included quarterly follow-ups (every 3 months), during which patients received guidance on the use of NBP and instructions on their baseline medications. This approach aimed to ensure proper medication adherence and minimize potential variability in diabetes management. Despite these efforts, the absence of specific glycemic control measures limits our ability to fully assess the impact of diabetes management on cognitive outcomes. Future studies should incorporate detailed measures of glycemic control (e.g., HbA1c levels) to better understand this relationship and to validate our findings.

Few studies have examined how NBP affects distinct cognitive domains. Our previous work indicated significant improvements in orientation and language scores after 12 months of NBP therapy (Han et al., 2025). Similarly, Yan et al. reported that NBP improved naming, memory, attention, and language after just 1 month of treatment (Yan et al., 2017). However, these studies did not account for glycemic status. Given that diabetes is a critical covariate in cognitive decline, our stratified analysis provides novel insights. In diabetic patients, NBP appeared to mitigate decline in orientation, potentially due to its role in upregulating VEGF and inhibiting caspase-3-mediated apoptosis (Tan et al., 2022). Animal studies have also shown that NBP improves spatial memory and reduces escape latency in diabetic rats, supporting its positive effect on orientation-related functions (Tian et al., 2017).

Interestingly, prior studies have shown that diabetes-related cognitive impairment predominantly affects visuospatial ability, naming, language, and memory, with orientation and attention relatively preserved (Sumbul-Sekerci et al., 2025). Similarly, Gallucci et al. found that the most frequently affected domains in PSCI were language, episodic memory, and executive function, with less impact on spatial orientation (Gallucci et al., 2024). Our study is the first to report that in diabetic individuals, the cognitive benefit of NBP is concentrated in orientation, whereas in non-diabetic individuals, the improvement is observed in language. This phenomenon may be attributable to the fact that the MMSE language (9 points) and orientation (10 points) sub-scales yielded relatively low baseline scores in our low-education, rural cohort, while their wider score ranges provided greater scope for detectable change, making any improvement more readily observable. These findings are exploratory and require further mechanistic studies and larger sample sizes to confirm and elucidate the domain-specific effects.

Notably, the absence of significant changes in the memory and executive sub-domains observed in our trial was likely attributable to both the limited follow-up duration and the restricted score range of these subscales. Compared with the language and orientation items, the baseline scores of the memory and executive components were already close to the ceiling, leaving insufficient room to detect subtle intervention effects. In addition, diabetes-associated cognitive impairment preferentially affects the fronto-striatal circuitry and the parahippocampal region, manifesting as decreased executive efficiency and slowed information-processing speed (Fu et al., 2022; Moran et al., 2013). In contrast, hippocampal structure remains relatively preserved within the first 6 months after stroke unless chronic, severe hyperglycaemia is present (Chaturvedi et al., 2020). The neuroprotective effects of NBP are mediated by improved mitochondrial energy metabolism, up-regulation of VEGF dependent angiogenesis, and inhibition of caspase-3-mediated apoptosis (Abdoulaye and Guo, 2016). These mechanisms are most effective in salvaging the acutely compromised cortico-subcortical penumbra. However, executive dysfunction resulting from chronic small-vessel disease is characterized by extensive white-matter demyelination (Abdoulaye and Guo, 2016; Kalaria and Erkinjuntti, 2006). 12 months of oral NBP was insufficient to reverse established axonal damage, explaining the lack of demonstrable benefit in the executive sub-domains. Memory consolidation, which relies heavily on hippocampal plasticity, may require a more protracted or multi-modal intervention (e.g., combined cognitive training) before any measurable improvement emerges. In our cohort, stroke patients were enrolled during the sub-acute phase and the mean glycated hemoglobin of the diabetic subgroup at one-year follow-up was 7.2%. This degree of glycaemic control, together with the relatively short observation window, had probably not yet produced advanced microvascular hippocampal sclerosis; consequently, no significant additional deterioration was observed in the memory sub-domain, and a putative “rescue” effect of NBP could not be detected.

Beyond the effect of NBP treatment, age and education level are well-recognized predictors of cognitive decline. In our analysis, we found that age ≥60 years was associated with an increased risk of cognitive decline in non-diabetic patients, while each additional year of education was associated with a reduced risk. This highlights the importance of including these demographic factors in our regression models to ensure an accurate assessment of NBP’s efficacy. However, these results were not significant in the diabetic subgroup. This may be due to the complex internal environment associated with the disease state of diabetes, or it may be attributed to the smaller sample size, which limited our ability to fully detect specific relationships. BMI, hypertension, and hyperlipidemia are also important factors that may influence cognitive outcomes (Chen et al., 2022; Hou et al., 2019). Although these factors were included in our multivariate models, their specific impact on the efficacy of NBP in diabetic and non-diabetic patients warrants further investigation. Given the established role of diabetes in cognitive decline and its potential interaction with the mechanisms of NBP, we considered diabetes status as a potential effect modifier in our study. Our subgroup analysis showed differential effects of NBP in diabetic and non-diabetic patients, suggesting that diabetes may indeed modify the therapeutic response to NBP. Future studies should explore other potential effect modifiers, such as genetic predispositions, lifestyle factors (e.g., smoking, alcohol use), and comorbid conditions (e.g., cardiovascular disease), to provide a more comprehensive understanding of the factors influencing NBP’s efficacy.

This study has several limitations that should be acknowledged. First, a phased cluster sampling strategy was employed rather than a fully randomized design. Although this sequential design facilitated field implementation in a rural setting and minimized treatment contamination, it may introduce temporal trends and selection bias. Inclusion of “study period” as a covariate in univariate models showed no significant effect on cognitive outcomes; nevertheless, residual bias cannot be fully excluded, and future studies should employ cluster or individual randomization for definitive validation. Second, self-reported schooling may misclassify MMSE thresholds and inflate baseline risk. Given the rural, low-literacy setting, we applied education-specific Chinese norms and validated findings with a binary “cognitive impairment” outcome that aligned with continuous ΔMMSE. Third, our cognitive impairment assessment used only the MMSE, which may not fully capture functions of the frontal, parietal, and occipital lobes. Despite its limitations, the MMSE is widely used in community settings due to its feasibility, especially among populations with lower education. Future studies should consider incorporating more comprehensive neuropsychological assessment tools, such as the Montreal Cognitive Assessment (MoCA) and the Addenbrooke’s Cognitive Examination (ACE), to better capture executive and visuospatial functions. Fourth, cognitive-modifying factors such as diet, physical activity, social engagement, stroke severity as measured by the National Institutes of Health Stroke Scale (NIHSS), neuroimaging metrics, and mood disorders, together with concomitant medications not related to stroke, were not assessed. Given that participants were enrolled during the sub-acute phase (1–6 months post-stroke) and remained functionally independent, NIHSS shows only a weak correlation with long-term cognition; we therefore used the modified Rankin Scale (mRS), blood pressure, body-mass index and lipid profile as partial surrogates of vascular burden. In future work, we will employ standardized questionnaires (International Physical Activity Questionnaire, IPAQ; Food Frequency Questionnaire, FFQ) and quantitative neuroimaging to capture these domains comprehensively, and will include comorbidities such as atrial fibrillation and hepatic or renal insufficiency to explore potential drug–disease interactions that may modify the effect of NBP. Fifth, diabetes was ascertained at baseline using HbA1c ≥ 6.5%, fasting glucose, or prior diagnosis, with blood drawn in the subacute stroke phase to avoid acute stress hyperglycaemia misclassification. Absence of 12-month HbA1c data constrains causal inference. Although continuous monitoring was not mandated in this observational cohort, it is now designated as a required refinement for future studies to strengthen result robustness. Moreover, although the overall sample (*n* = 594) satisfied the a-priori power requirement, the number of incident cognitive-decline events within each diabetes stratum remained limited, diminishing statistical precision. Consequently, the reported subgroup differences should be interpreted as exploratory and must be validated in adequately powered, multicenter trials specifically designed around event-driven sample-size calculations. Finally, the study was conducted in a single rural cohort from Jizhou District, Tianjin; caution is needed when extrapolating the findings to other ethnicities or urban settings. Yet this focus also represents a deliberate strength—providing the first locally relevant evidence on NBP efficacy among underserved, high-risk rural populations. Multi-center, multi-ethnic trials are warranted to confirm these observations.

## 5 Conclusion

In this prospective cohort study, we found that NBP treatment over a 12-month period was associated with a reduced risk of cognitive decline in IS survivors, with more pronounced benefits observed in non-diabetic patients. Moreover, the cognitive domains affected by NBP varied by glycemic status: orientation ability was beneficial to in diabetic patients, whereas language function was better preserved in non-diabetic individuals. It should be noted that this study is exploratory in nature, aiming to generate hypotheses for future research rather than to confirm causal relationships. Although the study is observational and cannot determine a causal association, these findings still provide novel evidence for the differential efficacy of NBP across various patient subgroups and underscore the potential value of early intervention during the subacute phase of ischemic stroke, which may maximize the neuroprotective effects of NBP.

Our results underscore the importance of considering metabolic comorbidities, such as diabetes, when evaluating neuroprotective interventions. For non-diabetic patients with subacute ischemic stroke and no prestroke cognitive impairment, adding NBP for 12 months on top of standard secondary prevention may be considered to reduce the risk of cognitive decline. In stroke survivors with diabetes, NBP has not demonstrated a clear overall protective effect against global cognitive deterioration, although a modest benefit on orientation was observed. Optimizing glycemic and metabolic control should take priority, and the decision to add NBP should be individualized under fully informed consent. These recommendations are preliminary evidence-based suggestions, given the single-center, observational nature of this study.

These findings warrant further validation in larger, multicenter randomized controlled trials and an exploration of their underlying mechanisms. Such efforts will further clarify the role of NBP in cognitive protection and support its integration into tailored post-stroke rehabilitation programs.

## Data Availability

The raw data supporting the conclusions of this article will be made available by the authors without undue reservation.

## Glossary

PSCI: post-stroke cognitive impairment
IS: ischemic stroke
DALYs: disability-adjusted life years
NBP: DL-3-n-butylphthalide
MMSE: mini-mental state examination
ΔMMSE: absolute change in MMSE score
SD: standard deviations
IQR: interquartile range
RR: risk ratio
CI: confidence interval
MRI: magnetic resonance imaging
BMI: body mass index
WC: waist circumference
HbA1c: glycosylated hemoglobin
FBG: fasting blood glucose
TC: total cholesterol
TG: triglycerides
HDL: high-density lipoprotein
LDL: low-density lipoprotein
Hcy: homocysteine
hs-CRP: high-sensitivity C-reactive protein
BP: Blood pressure
SBP: Systolic blood pressure
DBP: Diastolic blood pressure
OGTT: oral glucose tolerance test
VEGF: vascular endothelial growth factor
AGEs: advanced glycation end-products
BBB: blood–brain barrier.
