# Supplementary material for: Influence of diabetes on the efficacy of DL-3-n-butylphthalide in post-stroke cognitive impairment: a 12-month prospective cohort study
Source: Front Aging Neurosci. 2025 Nov 5;17:1649248. doi: 10.3389/fnagi.2025.1649248 (PMC12627063; doi:10.3389/fnagi.2025.1649248)
Supplement: Supplementary file 1 [file Table_1.DOCX]

Supplementary Material

**Supplemental Table 1**.Univariate analysis of risk factors of differences in Orientation and Language D-value and percentage in diabetics.

| Characteristics | Orientation  D-value | P value | Orientation D-value percentage | P value | Language D-value | P value | Language  D-value percentage | P value |
| --- | --- | --- | --- | --- | --- | --- | --- | --- |
| Treatment, n(%) |  | **0.042** |  | 0.059 |  | 0.104 |  | 0.158 |
| Butylphthalide | 0.05(1.76) |  | -0.01(0.22) |  | 0.10(1.20) |  | 0.003(0.17) |  |
| Control | 0.60(2.32) |  | 0.06(0.27) |  | 0.39(1.56) |  | 0.04(0.23) |  |
| Gender,n(%) |  | 0.272 |  | 0.349 |  | 0.691 |  | 0.873 |
| Men | 0.20(1.79) |  | 0.01(0.23) |  | 0.26(1.39) |  | 0.02(0.20) |  |
| Women | 0.59(2.63) |  | 0.05(0.29) |  | 0.18(1.39) |  | 0.02(0.21) |  |
| Age, years | 0.04(0.01,0.07) | **0.020** | 0.004(0.001,0.01) | **0.023** | -0.002(-0.02,0.02) | 0.845 | -0.002(-0.003,0.003) | 0.873 |
| Age group,n (%) |  | **0.015** |  | **0.016** |  | 0.414 |  | 0.375 |
| ＜60 years | -0.06(1.61) |  | -0.02(0.20) |  | 0.15(1.37) |  | 0.01(0.19) |  |
| ≥60 years | 0.56(2.29) |  | 0.05(0.27) |  | 0.30(1.40) |  | 0.03(0.21) |  |
| Education years, years | -0.08(-0.16,-0.004) | **0.039** | -0.01(-0.02,0.002) | 0.114 | -0.02(-0.08,0.03) | 0.360 | -0.003(-0.01,0.01) | 0.452 |
| Educational level, n(%) |  | 0.352 |  | 0.483 |  | 0.849 |  | 0.909 |
| Illiterate | 1.60(4.58) |  | 0.16(0.55) |  | 0.27(1.71) |  | 0.02(0.29) |  |
| Primary school | 0.31(2.20) |  | 0.01(0.27) |  | 0.34(1.49) |  | 0.03(0.23) |  |
| Junior school | 0.11(1.51) |  | 0.01(0.12) |  | 0.16(1.42) |  | 0.01(0.18) |  |
| High school and above | 0.30(2.06) |  | 0.03(0.12) |  | 0.18(0.84) |  | 0.02(0.11) |  |
| WHR | -1.32(-0.42,1.86) | 0.415 | -0.15(-0.52,0.23) | 0.450 | -0.05(-2.19,2.09) | 0.962 | -0.03(-0.33,0.28) | 0.869 |
| BMI, kg/m² | -0.05(-0.13,0.03) | 0.193 | -0.01(-0.02,0.003) | 0.194 | -0.04(-0.10,0.01) | 0.110 | -0.01(-0.02,0.004) | 0.063 |
| BMI groups, n (%) |  | 0.173 |  | 0.184 |  | **0.015** |  | **0.027** |
| Normal or underweight | -0.03(1.45) |  | -0.01(-0.07,0.05) |  | 0.13(1.04) |  | 0.02(0.15) |  |
| Overweight | 0.58(2.27) |  | 0.06(0.25) |  | 0.51(1.48) |  | 0.06(0.20) |  |
| Obesity | 0.12(1.97) |  | -0.001(0.26) |  | -0.04(1.33) |  | -0.02(0.21) |  |
| Smoking status, n (%) |  | 0.481 |  | 0.601 |  | 0.632 |  | 0.711 |
| Never smoking | 0.37(2.20) |  | 0.03(0.27) |  | 0.37(1.61) |  | 0.04(0.22) |  |
| Current smoking | 0.48(2.32) |  | 0.04(0.26) |  | 0.27(1.53) |  | 0.03(0.22) |  |
| Ever smoking | 0.12(1.72) |  | 0.01(0.22) |  | 0.14(1.11) |  | 0.01(0.16) |  |
| Drinking status, n (%) |  | 0.287 |  | 0.465 |  | 0.769 |  | 0.838 |
| Never drinking | -0.03(1.47) |  | -0.02(0.18) |  | 0.27(1.07) |  | 0.03(0.14) |  |
| Current drinking | 0.50(2.35) |  | 0.04(0.26) |  | 0.30(1.53) |  | 0.03(0.22) |  |
| Ever drinking | 0.21(1.89) |  | 0.02(0.25) |  | 0.16(1.32) |  | 0.01(0.19) |  |
| Hypertension, n (%) |  | 0.424 |  | 0.525 |  | **0.025** |  | **0.027** |
| No | 0.10(1.67) |  | 0.004(0.19) |  | 0.63(1.41) |  | 0.08(0.18) |  |
| Yes | 0.37(2.16) |  | 0.03(0.26) |  | 0.14(1.36) |  | 0.01(0.20) |  |
| Hyperlipidemia,n(%) |  | **0.036** |  | 0.980 |  | 0.320 |  | 0.472 |
| No | 0.33(2.08) |  | 0.02(0.25) |  | 0.15(1.23) |  | 0.01(0.19) |  |
| Yes | 0.29(2.06) |  | 0.02(0.24) |  | 0.33(1.52) |  | 0.03(0.21) |  |
| CHD, n (%) |  | **0.037** |  | **0.007** |  | 0.598 |  | 0.632 |
| No | 0.15(1.86) |  | 0.01(0.23) |  | 0.26(1.43) |  | 0.02(0.20) |  |
| Yes | 1.27(2.88) |  | 0.13(0.30) |  | 0.12(1.05) |  | 0.01(0.17) |  |
| SBP,mmHg | 0.01(-0.01,0.02) | 0.299 | 0.001(-0.001,0.002) | 0.385 | -0.001(-0.01,0.01) | 0.790 | -0.0002(-0.001,0.001) | 0.786 |
| DBP,mmHg | -0.01(-0.03,0.01) | 0.390 | -0.001(-0.004,0.001) | 0.345 | 0.003(-0.01,0.02) | 0.657 | 0.001(-0.002,0.003) | 0.636 |
| FBG,mmol/L | -0.03(-0.14,0.07) | 0.5113 | -0.003(-0.02,0.01) | 0.586 | 0.01(-0.06,0.08) | 0.864 | -0.0001(-0.01,0.01) | 0.980 |
| TC,mmol/L | -0.13(-0.38,0.11) | 0.284 | -0.02(-0.05,0.01) | 0.184 | -0.13(-0.29,0.03) | 0.119 | -0.01(-0.04,0.01) | 0.344 |
| TG,mmol/L | 0.01(-0.23,0.25) | 0.930 | 0.0004(-0.03,0.03) | 0.978 | -0.21(-0.37,-0.05) | **0.009** | -0.03(-0.05,-0.01) | **0.012** |
| HDL,mmol/L | 1.54(0.56,2.53) | **0.002** | 0.18(0.06,0.30) | **0.003** | 0.55(-0.12,1.22) | 0.109 | 0.10(0.003,0.20) | **0.043** |
| LDL,mmol/L | -0.39(-0.71,-0.07) | **0.017** | -0.05(-0.09,-0.01) | **0.008** | -0.14(-0.35,0.08) | 0.204 | -0.01(-0.04,0.02) | 0.472 |
| Hcy,mmol/L | -0.01(-0.03,0.01) | 0.435 | -0.001(-0.003,0.002) | 0.629 | -0.001(-0.02,0.02) | 0.940 | -0.0003(-0.003,0.002) | 0.787 |
| Hs-CRP,mg/L | -0.02(-0.06,0.02) | 0.290 | -0.003(-0.01,0.002) | 0.283 | -0.01(-0.03,0.02) | 0.560 | -0.001(-0.001,0.003) | 0.721 |

Note:(1)Bold fonts indicate P < 0.05, with significant differences in statistical results.

1. MMSE D-value and percentage are expressed as mean (SD) or β(95%CI).

(3)WHR Waist-to-Hip Ratio, BMI body mass index, CHD coronary heart disaese, SBP systolic blood pressure, DBP diastolic blood pressure, FPG fasting plasma glucose, TG triglycerides, TC total cholesterol, HDL High density lipoprotein, LDL Low density lipoprotein, Hcy homcysteine, hs-CRP high-sensitivity C-reactive protein, MMSE Mini-Mental State Examination, mRS modified Rankin Scale.

**Supplemental Table 2**.Univariate analysis of risk factors of differences in Orientation and Language D-value and percentage in non-diabetics.

| Characteristics | Orientation  D-value | P value | Orientation  D-value percentage | P value | Language D-value | P value | Language  D-value percentage | P value |
| --- | --- | --- | --- | --- | --- | --- | --- | --- |
| Treatment, n(%) |  | 0.053 |  | 0.084 |  | **0.013** |  | **0.015** |
| Butylphthalide | 0.01(1.36) |  | -0.01(0.19) |  | 0.00(1.09) |  | -0.01(0.16) |  |
| Control | 0.36(2.03) |  | 0.03(0.25) |  | 0.30(1.22) |  | 0.03(0.17) |  |
| Gender,n(%) |  | 0.131 |  | 0.334 |  | 0.829 |  | 0.900 |
| Men | 0.08(1.46) |  | 0.0003(0.18) |  | 0.14(1.10) |  | 0.01(0.15) |  |
| Women | 0.43(2.22) |  | 0.03(0.28) |  | 0.17(1.31) |  | 0.01(0.20) |  |
| Age, years | 0.03(0.01,0.05) | **0.013** | 0.003(0.0002,0.006) | **0.035** | 0.02(0.01,0.03) | **0.008** | 0.003(0.001,0.005) | **0.012** |
| Age group,n (%) |  | 0.255 |  | 0.434 |  | 0.071 |  | 0.101 |
| ＜60 years | 0.06(1.35) |  | -0.001(0.17) |  | 0.02(1.02) |  | -0.01(0.14) |  |
| ≥60 years | 0.26(1.95) |  | 0.02(0.25) |  | 0.24(1.24) |  | 0.02(0.18) |  |
| Education years, years | -0.08(-0.13,-0.03) | **0.002** | -0.01(-0.01,-0.001) | **0.021** | -0.01(-0.05,0.02) | 0.418 | -0.002(-0.007,0.003) | 0.412 |
| Educational level, n(%) |  | **0.028** |  | 0.079 |  | 0.604 |  | 0.621 |
| Illiterate | 1.40(3.33) |  | 0.13(0.37) |  | 0.50(1.82) |  | 0.07(0.30) |  |
| Primary school | 0.37(2.11) |  | 0.02(0.28) |  | 0.16(1.36) |  | 0.01(0.19) |  |
| Junior school | -0.17(0.91) |  | -0.02(0.12) |  | 0.15(0.91) |  | 0.01(0.12) |  |
| High school and above | 0.21(1.30) |  | 0.02(0.14) |  | 0.03(0.95) |  | -0.004(0.13) |  |
| WHR | -0.04(-0.39,0.30) | 0.807 | -0.004(-0.05,0.04) | 0.864 | 0.05(-0.18,0.28) | 0.659 | 0.01(-0.03,0.04) | 0.736 |
| BMI, kg/m² | 0.01(-0.05,0.06) | 0.814 | 0.001(-0.01,0.001) | 0.789 | -0.01(-0.05,0.02) | 0.528 | -0.001(-0.006,0.004) | 0.602 |
| BMI groups, n (%) |  | 0.882 |  | 0.915 |  | 0.849 |  | 0.859 |
| Normal or underweight | 0.19(1.71) |  | 0.01(0.20) |  | 0.18(1.10) |  | 0.01(0.16) |  |
| Overweight | 0.22(1.86) |  | 0.01(0.23) |  | 0.12(1.33) |  | 0.007(0.19) |  |
| Obesity | 0.12(1.57) |  | 0.001(0.23) |  | 0.19(0.92) |  | 0.02(0.12) |  |
| Smoking status, n (%) |  | 0.187 |  | 0.420 |  | 0.865 |  | 0.827 |
| Never smoking | 0.08(1.33) |  | -0.004(0.18) |  | 0.21(0.96) |  | 0.02(0.13) |  |
| Current smoking | 0.38(2.02) |  | 0.03(0.26) |  | 0.13(1.24) |  | 0.01(0.19) |  |
| Ever smoking | 0.04(1.64) |  | -0.003(0.20) |  | 0.14(1.22) |  | 0.01(0.16) |  |
| Drinking status, n (%) |  | 0.544 |  | 0.648 |  | 0.413 |  | 0.430 |
| Never drinking | 0.01(1.32) |  | -0.01(0.17) |  | 0.31(1.16) |  | 0.03(0.16) |  |
| Current drinking | 0.27(2.05) |  | 0.02(0.27) |  | 0.09(1.21) |  | 0.003(0.18) |  |
| Ever drinking | 0.16(1.50) |  | 0.01(0.17) |  | 0.15(1.10) |  | 0.01(0.15) |  |
| Hypertension, n (%) |  | 0.900 |  | 0.619 |  | 0.072 |  | 0.670 |
| No | 0.17(1.83) |  | -0.0001(0.23) |  | 0.20(1.37) |  | 0.02(0.20) |  |
| Yes | 0.19(1.70) |  | 0.01(0.22) |  | 0.13(1.07) |  | 0.01(0.15) |  |
| Hyperlipidemia,n(%) |  | **0.005** |  | **0.017** |  | 0.704 |  | 0.817 |
| No | 0.37(1.89) |  | 0.03(0.24) |  | 0.13(1.18) |  | 0.01(0.17) |  |
| Yes | -0.12(1.41) |  | -0.03(0.17) |  | 0.18(1.13) |  | 0.01(0.16) |  |
| CHD, n (%) |  | 0.674 |  | 0.513 |  | 0.110 |  | 0.582 |
| No | 0.17(1.78) |  | 0.01(0.23) |  | 0.16(1.16) |  | 0.01(0.16) |  |
| Yes | 0.30(1.35) |  | 0.03(0.15) |  | 0.05(1.22) |  | -0.002(0.18) |  |
| SBP,mmHg | 0.01(0.01,0.02) | **0.001** | 0.002(0.001,0.003) | **＜0.001** | 0.01(0.01,0.02) | **＜0.001** | 0.001(0.001,0.002) | **＜0.001** |
| DBP,mmHg | 0.02(0.003,0.04) | **0.019** | 0.002(0.0005,0.005) | **0.016** | 0.02(0.01,0.03) | **＜0.001** | 0.002(0.001,0..004) | **0.002** |
| FBG,mmol/L | 0.21(-0.12,0.55) | 0.215 | 0.02(-0.02,0.06) | 0.319 | 0.11(-0.12,0.33) | 0.352 | 0.01(-0.02,0.05) | 0.397 |
| TC,mmol/L | 0.14(-0.03,0.31) | 0.098 | 0.02(-0.01,0.04) | 0.176 | 0.11(-0.004,0.22) | 0.060 | 0.01(-0.003,0.03) | 0.110 |
| TG,mmol/L | -0.04(-0.19,0.11) | 0.575 | -0.01(-0.03,0.01) | 0.415 | 0.01(-0.09,0.11) | 0.796 | 0.002(-0.01,0.02) | 0.766 |
| HDL,mmol/L | 0.23(-0.40,0.86) | 0.468 | 0.04(-0.04,0.12) | 0.349 | 0.14(-0.28,0.56) | 0.506 | 0.02(-0.04,0.08) | 0.538 |
| LDL,mmol/L | 0.17(-0.03,0.37) | 0.101 | 0.02(-0.01,0.04) | 0.175 | 0.13(-0.01,0.26) | 0.059 | 0.02(-0.004,0.03) | 0.121 |
| Hcy,mmol/L | 0.004(-0.01,0.02) | 0.597 | 0.001(-0.001,0.003) | 0.414 | 0.01(0.001,0.02) | **0.037** | 0.001(0.000,0.003) | 0.051 |
| Hs-CRP,mg/L | -0.01(-0.05,0.04) | 0.822 | -0.00(-0.06,0.06) | 0.984 | -0.01(-0.04,0.02) | 0.521 | -0.001(-0.006,0.003) | 0.557 |

Note:(1)Bold fonts indicate P < 0.05, with significant differences in statistical results.

(2)Orientation and Language D-value and percentage are expressed as mean(SD) or β(95%CI).

(3)WHR Waist-to-Hip Ratio, BMI body mass index, CHD coronary heart disaese, SBP systolic blood pressure, DBP diastolic blood pressure, FPG fasting plasma glucose, TG triglycerides, TC total cholesterol, HDL High density lipoprotein, LDL Low density lipoprotein, Hcy homcysteine, hs-CRP high-sensitivity C-reactive protein, MMSE Mini-Mental State Examination, mRS modified Rankin Scale.

**Supplemental Table 3**.Univariate analysis of risk factors for other cognitive domain scores in diabetics and non-diabetics..

| Characteristics | Diabetics | | | | | | Non-diabetics | | | | | |
| --- | --- | --- | --- | --- | --- | --- | --- | --- | --- | --- | --- | --- |
|  | Attention and calculation，  mean(SD) | P value | Memory，  mean(SD) | P value | Recall，  mean(SD) | P value | Attention and calculation，  mean(SD) | P value | Memory，  mean(SD) | P value | Recall，  mean(SD) | P value |
| Treatment, n(%) |  | 0.836 |  | 0.205 |  | 0.509 |  | 0.230 |  | 0.678 |  | 0.290 |
| Butylphthalide | 0.17(1.53) |  | 0.03(0.46) |  | 0.11(1.18) |  | 0.12(1.49) |  | -0.07(0.96) |  | 0.09(0.67) |  |
| Control | 0.21(1.58) |  | 0.13(0.72) |  | 0.21(1.12) |  | 0.32(1.71) |  | -0.03(1.09) |  | 0.03(0.37) |  |
| Gender,n(%) |  | 0.220 |  | 0.057 |  | **0.029** |  | 0.172 |  | 0.995 |  | 0.697 |
| Men | 0.28(1.41) |  | 0.02(0.49) |  | 0.06(1.34) |  | 0.30(1.57) |  | 0.06(0.56) |  | -0.06(1.03) |  |
| Women | -0.03(1.84) |  | 0.23(0.80) |  | 0.42(1.15) |  | 0.05(1.67) |  | 0.06(0.51) |  | -0.02(1.01) |  |
| Age, years |  | 0.961 |  | 0.138 |  | 0.332 |  | 0.167 |  | **0.036** |  | 0.053 |
| Age group,n (%) |  | 0.937 |  | **0.010** |  | 0.250 |  | 0.144 |  | 0.265 |  | **0.028** |
| ＜60 years | 0.20(1.63) |  | -0.02(0.15) |  | 0.06(0.89) |  | 0.07(1.47) |  | 0.03(0.38) |  | -0.20(0.91) |  |
| ≥60 years | 0.19(1.50) |  | 0.15(0.76) |  | 0.23(1.29) |  | 0.32(1.68) |  | 0.09(0.63) |  | 0.05(1.09) |  |
| Education years, years |  | 0.616 |  | **＜0.001** |  | 0.911 |  | 0.440 |  | **0.042** |  | 0.061 |
| Educational level, n(%) |  | 0.124 |  | **0.010** |  | 0.479 |  | 0.323 |  | 0.355 |  | 0.138 |
| Illiterate | -0.33(1.29) |  | 0.80(1.21) |  | 0.60(1.45) |  | 0.55(1.76) |  | 0.30(1.08) |  | 0.15(1.04) |  |
| Primary school | 0.33(1.76) |  | 0.03(0.60) |  | 0.13(1.25) |  | 0.13(1.79) |  | 0.10(0.67) |  | -0.01(1.16) |  |
| Junior school | 0.30(1.52) |  | 00.05(0.50) |  | 0.09(1.02) |  | 0.36(1.67) |  | 0.02(0.39) |  | 0.01(0.98) |  |
| High school and above | -0.15(1.08) |  | -0.02(0.16) |  | 0.25(1.08) |  | 0.01(0.84) |  | 0.01(0.12) |  | -0.30(0.74) |  |
| WHR |  | 0.390 |  | 0.323 |  | 0.209 |  | 0.849 |  | 0.842 |  | 0.956 |
| BMI, kg/m² |  | 0.763 |  | 0.910 |  | 0.834 |  | 0.642 |  | 0.377 |  | 0.303 |
| BMI groups, n (%) |  | 0.744 |  | 0.922 |  | 0.897 |  | 0.523 |  | 0.210 |  | 0.204 |
| Normal or underweight | 0.00(1.63) |  | 0.09(0.59) |  | 0.16(1.05) |  | 0.38(1.74) |  | 0.04(0.68) |  | -0.04(1.01) |  |
| Overweight | 0.24(1.39) |  | 0.06(0.58) |  | 0.13(1.29) |  | 0.17(1.60) |  | 0.12(0.60) |  | 0.04(1.05) |  |
| Obesity | 0.20(1.70) |  | 0.10(0.63) |  | 0.20(1.02) |  | 0.15(1.48) |  | 0.00(0.20) |  | -0.19(0.97) |  |
| Smoking status, n (%) |  | 0.497 |  | 0.464 |  | 0.600 |  | 0.521 |  | 0.595 |  | 0.197 |
| Never smoking | 0.43(1.50) |  | 0.07(0.68) |  | 0.24(1.16) |  | 0.31(1.42) |  | 0.11(0.64) |  | 0.04(1.10) |  |
| Current smoking | 0.13(1.69) |  | 0.14(0.71) |  | 0.22(1.16) |  | 0.10(1.71) |  | 0.04(0.51) |  | 0.01(1.00) |  |
| Ever smoking | 0.13(1.43) |  | 0.03(0.42) |  | 0.07(1.14) |  | 0.29(1.61) |  | 0.06(0.50) |  | -0.18(1.00) |  |
| Drinking status, n (%) |  | 0.672 |  | 0.304 |  | 0.332 |  | 0.669 |  | 0.848 |  | 0.420 |
| Never drinking | 0.39(1.54) |  | -0.03(0.31) |  | 0.00(1.39) |  | 0.33(1.48) |  | 0.09(0.48) |  | 0.09(1.00) |  |
| Current drinking | 0.20(1.76) |  | 0.14(0.65) |  | 0.28(1.05) |  | 0.14(1.61) |  | 0.07(0.54) |  | -0.06(1.06) |  |
| Ever drinking | 0.12(1.29) |  | 0.05(0.61) |  | 0.09(1.16) |  | 0.26(1.65) |  | 0.04(0.57) |  | -0.11(1.00) |  |
| Hypertension, n (%) |  | 0.883 |  | 0.249 |  | 0.406 |  | 0.073 |  | 0.141 |  | 0.986 |
| No | 0.16(1.61) |  | 0.02(0.32) |  | 0.04(1.41) |  | 0.27(1.68) |  | 0.15(0.73) |  | -0.05(1.11) |  |
| Yes | 0.20(1.54) |  | 0.10(0.65) |  | 0.19(1.07) |  | 0.20(1.57) |  | 0.03(0.44) |  | -0.05(0.99) |  |
| Hyperlipidemia,n(%) |  | 0.457 |  | 0.428 |  | 0.290 |  | 0.460 |  | **＜0.001** |  | 0.516 |
| No | 0.27(1.78) |  | 0.11(0.70) |  | 0.24(1.09) |  | 0.17(1.69) |  | 0.13(0.63) |  | -0.02(1.04) |  |
| Yes | 0.12(1.28) |  | 0.05(0.48) |  | 0.08(1.20) |  | 0.30(1.46) |  | -0.04(0.32) |  | -0.09(1.01) |  |
| CHD, n (%) |  | 0.258 |  | 0.920 |  | 0.553 |  | 0.441 |  | 0.270 |  | 0.066 |
| No | 0.24(1.56) |  | 0.08(0.61) |  | 0.14(1.14) |  | 0.24(1.57) |  | 0.05(0.51) |  | -0.08(1.03) |  |
| Yes | -0.09(1.47) |  | 0.09(0.52) |  | 0.27(1.23) |  | 0.03(1.88) |  | 0.19(0.74) |  | 0.24(0.93) |  |
| SBP,mmHg |  | 0.392 |  | 0.998 |  | 0.079 |  | 0.710 |  | 0.684 |  | 0.966 |
| DBP,mmHg |  | 0.431 |  | 0.353 |  | **0.020** |  | 0.397 |  | 0.418 |  | 0.120 |
| FBG,mmol/L |  | 0.301 |  | 0.715 |  | 0.651 |  | 0.136 |  | 0.104 |  | 0.452 |
| TC,mmol/L |  | 0.056 |  | 0.605 |  | 0.556 |  | 0.618 |  | 0.075 |  | 0.181 |
| TG,mmol/L |  | 0.923 |  | 0.476 |  | 0.575 |  | 0.832 |  | 0.745 |  | 0.751 |
| HDL,mmol/L |  | 0.595 |  | 0.056 |  | **0.002** |  | 0.993 |  | 0.256 |  | 0.817 |
| LDL,mmol/L |  | 0.073 |  | 0.689 |  | 0.800 |  | 0.631 |  | 0.123 |  | 0.082 |
| Hcy,mmol/L |  | 0.845 |  | 0.382 |  | 0.616 |  | 0.612 |  | 0.235 |  | 0.956 |
| Hs-CRP,mg/L |  | 0.473 |  | 0.449 |  | 0.335 |  | 0.308 |  | 0.700 |  | 0.720 |

Note:(1)Bold fonts indicate P < 0.05, with significant differences in statistical result.

(2)WHR Waist-to-Hip Ratio, BMI body mass index, CHD coronary heart disaese, SBP systolic blood pressure, DBP diastolic blood pressure, FPG fasting plasma glucose, TG triglycerides, TC total cholesterol, HDL High density lipoprotein, LDL Low density lipoprotein, Hcy homcysteine, hs-CRP high-sensitivity C-reactive protein, MMSE Mini-Mental State Examination, mRS modified Rankin Scale.
